# In Vitro Interaction of Doxorubicin-Loaded Silk Sericin Nanocarriers with MCF-7 Breast Cancer Cells Leads to DNA Damage

**DOI:** 10.3390/polym13132047

**Published:** 2021-06-22

**Authors:** Ionuț-Cristian Radu, Cătălin Zaharia, Ariana Hudiță, Eugenia Tanasă, Octav Ginghină, Minodora Marin, Bianca Gălățeanu, Marieta Costache

**Affiliations:** 1 Advanced Polymer Materials Group, Faculty of Applied Chemistry and Materials Science, University Politehnicaof Bucharest, 1-7 Gh. Polizu Street, Sector 1, 011061 Bucharest, Romania; radu.ionut57@yahoo.com (I.-C.R.); zaharia.catalin@gmail.com (C.Z.); minodora.marin@ymail.com (M.M.); 2 Department of Biochemistry and Molecular Biology, University of Bucharest, 050095 Bucharest, Romania; arianahudita@yahoo.com (A.H.); marietacostache@yahoo.com (M.C.); 3 University Politehnica of Bucharest, 313 Splaiul Independentei, Sector 6, 060042 Bucharest, Romania; eugenia.vasile27@gmail.com; 4 Department of Surgery, “Sf. Ioan” Clinical Emergency Hospital, 042122 Bucharest, Romania; octav.ginghina@umfcd.ro; 5 Faculty of Dental Medicine, Department II, “Carol Davila” University of Medicine and Pharmacy, 020021 Bucharest, Romania

**Keywords:** silk sericin, nanoprecipitation, breast cancer, doxorubicin

## Abstract

In this paper, *Bombyx mori* silk sericin nanocarriers with a very low size range were obtained by nanoprecipitation. Sericin nanoparticles were loaded with doxorubicin, and they were considered a promising tool for breast cancer therapy. The chemistry, structure, morphology, and size distribution of nanocarriers were investigated by Fourier transformed infrared spectroscopy (FTIR–ATR), scanning electron microscopy (SEM) and transmission electron microscopy (TEM), and dynamic light scattering (DLS). Morphological investigation and DLS showed the formation of sericin nanoparticles in the 25–40 nm range. FTIR chemical characterization showed specific interactions of protein–doxorubicin–enzymes with a high influence on the drug delivery process and release behavior. The biological investigation via breast cancer cell line revealed a high activity of nanocarriers in cancer cells by inducing significant DNA damage.

## 1. Introduction

Silks are natural protein-like fibers produced by arthropods, such as spiders or silkworms. Domestic-species-producing silks have been used since antiquity. Certain species, such as domesticated silkworm *Bombyx mori*, have a central role within textile industry applications and more recently in biomedical applications [1,2,3]. *Bombyx mori* proteins have been intensively studied for their biocompatibility, great mechanical properties, tunable biodegradation process, easy processing, and favorable source supply. Silk is composed of two major proteins: silk fibroin (fibrous protein) and silk sericin (globular protein) [4,5,6,7,8]. Silk fibroin is the main protein with large usage in the biomedical field. Silk sericin was originally removed as it was associated with the general immune body response to silks [9,10,11]. Therefore, silk sericin was largely neglected as a biomaterial for medical applications. However, silk sericin has attracted the particular interest of researchers due to special properties such as antioxidant effect, UV protection, moisture adsorption, or antibacterial protection. Recent studies in the literature revealed that sericin is currently used in cosmetics, pharmaceuticals, wound dressing, drug delivery, or cell culture. The antioxidant properties allow sericin to stand against lipid peroxidation by scavenging reactive species or to suppress tumor genesis by reducing oxidative stress or inflammatory responses [12,13,14]. Furthermore, the antioxidant properties of silk sericin contributed to cancer applications due to the capability to reduce oxidative stress or suppressing cancer cytokines for skin and colon cancer [15,16]. In recent years, silk sericin was used in the development of scaffolds for regenerative medicine in wound healing or tissue engineering [6,17,18,19].

In this regard, silk sericin may favor and sustain migration, proliferation, or collagen type I production due to methionine amino acid [20,21,22]. Silk sericin promoted open wound healing and added to silver-zinc sulfadiazine cream prevented burn wound infection. It was also effective in healing second-degree burn wounds without serious inflammatory reactions [22]. Besides particular properties, which recommended silk sericin for various biomedical applications, the drug-delivery field represents the specific area in which different nanoparticle systems have been developed [23,24,25,26,27,28,29,30]. However, the future concept pathway is used to overcome the current limitations so that it sustains the continuous development of nanoparticle systems. Silk sericin proved the ability for self-assembling capacity by loading various active principles. The unique chemistry favored surface charge modification for DNA binding and active targeting by poly(ethylene glycol) (PEG) and folate for cancer management [24,26,31].

This unique ability offers the possibility to easily prepare nanoparticles for drug/biomolecule delivery. These characteristics are tightly related to sericin chemistry and, in the last decades, studies showed new interesting insights on silk sericin structure. Therefore, silk sericin showed less amphiphilic character but higher hydrophilic character. This aspect was considered an impediment for self-assembling into nanoparticles, as compared to silk fibroin [32]. However, the synergistic effect of a proper preparation method, such as nanoprecipitation, and a specific precipitation agent may favor the preparation of silk sericin nanoparticles. Nanoparticles should display the desired characteristics such as size, morphology, and size distribution. Nanoprecipitation is a simple and fast method to produce nanoparticles from various types of polymers [33,34,35,36,37,38].

Doxorubicin is an anthracycline with therapeutic effects in a wide range of solid tumors, which still plays a major role in chemotherapy that induces apoptosis by causing DNA damage [39]. A great effort has been made to develop targeted nanodrug delivery systems due to their high therapeutic efficacy in cancer management. Polymeric nanoparticles are promising systems for drug delivery based on their nanometric size, high surface-area-to-volume ratio, favorable drug release profiles, and targeting features that can promote their preferential accumulation in tumor tissue. Various preparation routes have been addressed in the literature in an attempt to show the interaction of doxorubicin with various polymeric systems [40,41]. In this regard, doxorubicin-loaded RGD-conjugated polypeptide nanoformulation was developed by an emulsion solvent evaporation method [42]. These zwitterionic biodegradable drug-loaded polypeptide vesicles showed great potential for cancer treatment having high drug loading content (45%) and loading efficiency (95%) [42]. An interesting approach was related to the encapsulation of doxorubicin by polymerization-induced self-assembly methods [41]. Photopolymerization of various monomers in the presence of photocatalytic doxorubicin hydrochloride proved an interesting method to prepare drug-loaded polymeric nanoparticles with higher polymerization rates and good doxorubicin encapsulation efficiency [41]. Protein nanoparticles were also prepared by various routes to easily entrap and release various anticancer drugs [43]. They are prepared within biological systems, require fewer production steps, and show high biocompatibility and biodegradability, as compared to synthetic polymers. Protein nanoparticles have been prepared from various proteins including water-soluble proteins (bovine and human serum albumin, silk sericin) and partially soluble or insoluble proteins (silk fibroin, zein, and gliadin) [44]. TRAIL and Dox-loaded albumin nanoparticles showed great potential for synergistic apoptosis-based anticancer therapy [45].

This research study emphasizes the possibility to easily develop silk sericin nanoparticles by an optimized nanoprecipitation method. The novelty of the study arises from the development of sericin nanoparticles with a lower size distribution with respect to the literature data. Furthermore, the nanoparticles’ preparation is based on an optimized procedure that can be easily transferred being useful for other similar systems.

The present research study reports on the preparation and complex characterization of self-assembled silk sericin nanoparticles in the presence of acetone with secondary conformational changes. Doxorubicin loading in sericin nanoparticles and drug release behaviors were studied in neutral, acidic, and enzymatic media. DLS, SEM, and TEM were employed for morphological and structural characterization of the nanoparticles. The structural changes showed similar behavior to silk fibroin by revealing a special and stable physical crosslinking structure of nanoparticles. Moreover, we investigated the sericin particles’ potential to reduce MCF-7 cells’ viability and to induce DNA fragmentation when loaded with doxorubicin.

## 2. Materials and Methods

### 2.1. Materials

*Bombyx mori* silk sericin powder (quality level 200) and all other reagents, including sodium hydroxide (reagent grade, ≥98%, pellets, anhydrous), potassium phosphate (ACS reagent, ≥98%), hydrochloric acid (ACS reagent, 37%), doxorubicin (doxorubicin hydrochloride 98.0–102.0%, HPLC), protease XIV from *Streptomyces griseus* (≥3.5 units/mg solid), *α-Chymotrypsin* from bovine pancreas (≥40 units/mg protein), and nonsolvent acetone (ACS reagent, ≥99.5%) were provided by Sigma Aldrich (3050 SPRUCE Street, St. Louis, MO 63103, USA).

### 2.2. Preparation of Silk Sericin Nanoparticles

Firstly, silk sericin solutions were obtained by direct dissolution of protein in distilled water considering the hydrophilic character and good water solubility. Briefly, solutions with 0.1, 0.25, 0.5, and 1% (*w*/*v*) were prepared under moderate stirring at room temperature.

**Scheme 1:**
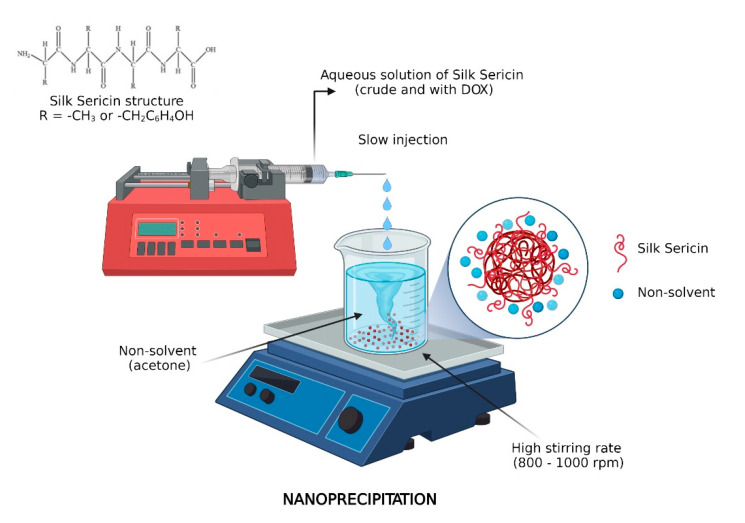
Workflow of sericin nanoparticle preparation.

Silk sericin nanoparticles were prepared via nanoprecipitation in which silk sericin solutions (0.1, 0.25, 0.5, and 1% *w*/*v*) were added into an organic phase of a nonsolvent, acetone. The nanoprecipitation technique involved the drop-wise addition of protein aqueous solution into acetone under vigorous stirring. The resulted nanoparticles were recovered by water and acetone vaporization. The workflow of nanoparticle preparation is shown in Scheme 1. The nanoparticle formulations were called SER 0.1%–SER 1% following the sericin concentration.

### 2.3. Drug Loading in Sericin Nanoparticles

Doxorubicin was loaded within sericin nanoparticles via the direct method by dissolution into the aqueous phase. The next step involved the addition of the dissolved drug aqueous phase into the acetone organic phase under vigorous stirring at neutral pH of 7.45 (1.25–5 wt.% doxorubicin in NPs). The drug-loaded nanoparticles were also recuperated by water and acetone vaporization. The drug was loaded in all prepared formulations 0.1, 0.25, 0.5, and 1% (*w*/*v*).

### 2.4. Drug Release Behavior

In vitro drug release behaviors of doxorubicin-loaded sericin nanoparticles were investigated in different pH conditions and enzymatic medium at 37 °C. Briefly, 5 mL of drug-loaded nanoparticles (4 wt.%) in phosphate buffer saline solution (PBS, pH 7.45) was placed in a tubular cellulose membrane, followed by immersion in flasks with a fixed volume (40 mL). The flasks were further incubated in an orbital mixer (Benchmark Scientific) at 300 rpm, and 37.0 ± 0.5 °C. 5 mL of PBS dialysate was collected at predetermined time intervals and then investigated by UV–VIS spectroscopy (SHIMADZU UV-3600 instrument). To maintain a constant volume, after each collection, 5 mL of fresh PBS were added to every flask. A similar procedure was performed for an acidic medium (pH = 3). Protease type XIV from *Streptomyces griseus* and protease α-chymotrypsin from bovine pancreas were used for enzymatic drug release test. Enzymes were added in a suspension of drug-loaded NPs for 1 h prior to release test, and the drug release behavior followed the above-mentioned protocol. The enzyme activity was 8 U/mL. The release efficiency was calculated as follows:

(1) $$RE\;\left(\%\right)=\frac{amount\;of\;released\;DOX}{amount\;of\;the\;loaded\;DOX\;}\times 100$$

The encapsulation efficiency was calculated with the following equation:

(2) $$EE\;\left(\%\right)=\frac{amount\;of\;the\;loaded\;DOX-amount\;of\;unloaded\;DOX}{amount\;of\;the\;loaded\;DOX\;}\times 100$$

### 2.5. Characterization Methods

#### 2.5.1. FTIR–ATR Analysis

FTIR–ATR investigation was performed using a Bruker Vertex 70 FTIR spectrophotometer with an attenuated total reflectance (ATR) accessory. FTIR spectrophotometer used 32 scans and a resolution of 4 cm^−1^ in mid-IR region 4000–600 cm^−1^. Sericin nanoparticles SER 1% (*w*/*v*), loaded with doxorubicin and aqueous mixtures of enzyme–doxorubicin, were analyzed.

#### 2.5.2. Morphological Characterization

Scanning electron microscopy (SEM) analysis was performed to reveal the main features of sericin nanoparticles, including aggregates’ size, shape, or morphology. Silk sericin nanoparticles were investigated by a Quanta Inspect F scanning electron microscopy device equipped with a field emission gun (FEG) with 1.2 nm resolution and with an X-ray energy-dispersive spectrometer (EDS). 

Transmission electron microscopy (TEM) analysis was performed using a TECNAI F30 G2 S-TWIN microscope operated at 300 kV in an energy-dispersive X-ray analysis (EDAX) facility. Formulations of sericin nanoparticles with 1% (*w*/*v*) and 0.1% (*w*/*v*) were evaluated by TEM.

#### 2.5.3. Dynamic Light Scattering (DLS)

The size distribution of the nanoparticles was evaluated by dynamic light scattering in a static domain using a Malvern Zetasizer Nano instrument. Nanoparticles prepared from all sericin concentrations were subjected to DLS investigation (0.1, 0.25, 0.5, and 1% (*w*/*v*)).

The average molecular weight of silk sericin was determined by DLS in a static domain using a Malvern Zetasizer Nano instrument in the molecular weight module. The analysis was performed using glass cuvettes with square aperture. Toluene was used as a reference standard solvent and water as a common solvent for silk sericin. Zeta potential and isoelectric point were also determined by DLS using several protein solutions with pH ranging between acid and neutral (pH 1–7.45). The zeta potential value was considered at a neutral point. The isoelectric point value was considered for zeta potential.

#### 2.5.4. Conformational Analysis by Circular Dichroism (CD)

The secondary structure of silk sericin solution and sericin nanoparticles dispersion was evaluated by a Jasco J-1500 spectrophotometer, Japan (J-1500 Circular Dichroism Spectrophotometer) using a quartz cell of 1 mm path length. During this analysis, the samples were scanned three times at low concentrations in the range of 180–250 nm with a scan rate of 100 nm/min.

### 2.6. In Vitro Biological Evaluation of Free and Doxorubicin-Loaded Sericin Nanocarriers

#### 2.6.1. Cell Culture Model

MCF-7 human epithelial mammary gland cell line (ATCC^®^ HTB-22™) was employed in this study as in vitro model since it retains several characteristics of differentiated mammary epithelium including the ability to process estradiol via cytoplasmic estrogen receptors and the capability of forming domes. Moreover, the MCF-7 cell line is positive for the estrogen and the progesterone receptor and negative for the HER2 marker. The MCF-7 cells were cultured in Dulbecco’s modified Eagle medium (DMEM) supplemented with 10% fetal bovine serum (FBS) and 1% antibiotic–antimycotic solution (ABAM, containing 100 U/mL penicillin, 100 µg/mL streptomycin, and 0.25 µg amphotericin B) and maintained at 37 °C in a humidified air atmosphere of 5% CO_2_ throughout the experiment. The medium renewal was carried out every other day.

#### 2.6.2. Cell Viability Assay

The MTT assay was used to measure MCF-7 breast cancer cells’ viability after incubation with Ser NPs and Ser NPs + DOX at a final concentration of 20 mg/mL Ser NPs ± Dox. Briefly, MCF-7 cells were seeded at an initial density of 2 × 10^5^ cells/cm^2^ in 96–well culture plates and treated with 20 mg/mL Ser NPs and Ser NPs + DOX for 6 and 24 h. At each time point, the culture media was discarded and replaced with a freshly prepared solution of MTT (1 mg/mL). The samples were further incubated for 4 h in standard cell culture conditions to allow the metabolically active cells to form formazan crystals, which were dissolved in DMSO. The absorbance of the resulting solutions was measured at 550 nm using a Flex Station III multimodal reader (Molecular Devices).

#### 2.6.3. Cytoskeleton Investigation and DAPI staining

To evaluate the potential morphological modifications induced in the MCF-7 breast cancer cells by the treatment with Ser NPs + DOX, the cytoskeleton’s actin filaments were stained with FITC–phalloidin. In this view, MCF-7 cells were seeded at an initial cell density of 2 × 10^5^ cells/cm^2^ in 96–well culture plates and treated with 20 mg/mL Ser NPs and Ser NPs + DOX for 6 and 24 h. At each time point, the MCF-7 monolayers were fixed with a 4% paraformaldehyde solution for 20 min, permeabilized with a 2% BSA/0.1% Triton X100 solution for 1 h and consequently stained with FITC–conjugated phalloidin for 1 h at 37 °C in a humidified environment. In the end, the MCF-7 monolayers were stained with DAPI to highlight cell nuclei and reveal chromatin fragmentation. The samples were imaged using the Olympus IX73 inverted fluorescence microscope (Olympus) and images were captured using CellSense Imaging Software.

#### 2.6.4. Measurement of DNA Damage by Comet Assay

The DNA damage induced by the Ser NPs + DOX in MCF-7 breast cancer cells was quantified at a single cell level using OxiSelect Comet Assay Kit three-well slides (Cell Biolabs) assay. The MCF-7 cells were seeded in six-well plates at an initial density of 0.3 × 10^6^ cells/cm^2^ and treated with simple Ser NPs and DOX-loaded Ser NPs. After 6 h and 24 h, the cells were detached from the culture vessels and processed as recommended by the manufacturers’ instructions. Briefly, cells were resuspended in agarose in a 1:10 ratio and transferred on the agarose precoated comet assay slides. After gelling, the comet assay slides were immersed in the lysis buffer for 60 min at 40 °C in the dark, followed by alkaline solution treatment for 30 min in the same conditions. Then, the slides were placed in the electrophoresis solution and then transferred to the electrophoresis tank. The electrophoresis was carried out at 1 V/cm for 15 min. Finally, samples were stained with 100 µL Vista Green DNA Dye solution for 15 min at room temperature in the dark. In total, 150 randomly selected cells from each slide were analyzed using a fluorescence microscope (Olympus IX73) and CellSense software. The length of the comet tail was chosen as an indicator of DNA damage.

#### 2.6.5. Statistical Analysis

The data obtained from the MTT assay were statistically analyzed using GraphPad Prism 6 software (San Diego, CA, USA), one-way ANOVA, and the Bonferroni test. Control samples were considered as 100% viability for each time point. All the experiments were performed with three biological replicates and each data set is presented as the average of three replicates (mean ± standard deviation). A value of *p* ≤ 0.05 was considered to indicate a statistically significant difference. All experimental controls were represented by MCF-7 cell cultures, where fresh culture media was added instead of nanoparticles treatment and were identically processed as described for each assay separately.

## 3. Results and Discussions

### 3.1. FTIR–ATR Analysis

**Figure 1:**
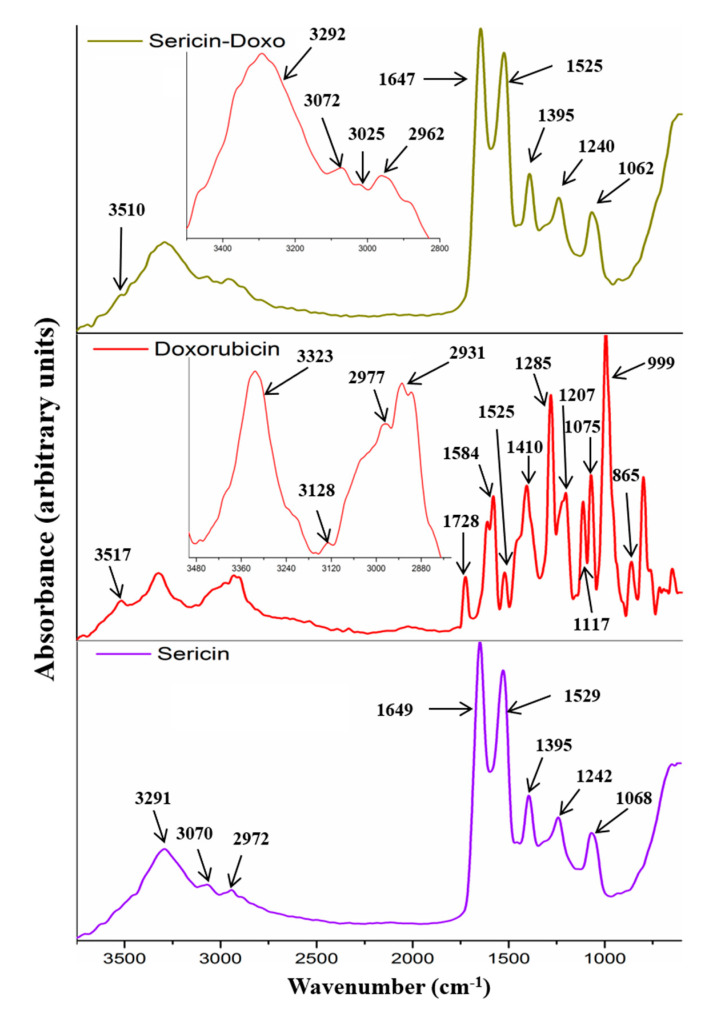
FTIR–ATR spectra of sericin, doxorubicin, and doxorubicin-loaded sericin nanoparticles.

FTIR investigation showed the main characteristic peaks for doxorubicin, enzymes, sericin, and their interactions. The spectrum of doxorubicin had the following peaks: peak at 3517 cm^−1^ was assigned to water molecules bonded within the drug structure; peak at 3323 cm^−1^ was attributed to hydroxyl stretching vibration; peak at 3128 cm^−1^ was attributed to N-H stretching vibration; peaks at 2977 cm^−1^ and 2931 cm^−1^ were assigned to C-H stretching vibration within the ring; peak at 1728 cm^−1^ was assigned to C=O stretching in carbonyl group within vibrating in quinone and ketone; peak at 1584 cm^−1^ was attributed to N-H bending vibration and C-N stretching vibration; peak at 1525 cm^−1^ was assigned to hydroxyl group bending vibration; peak at 1410 cm^−1^ was attributed to methyl bending vibration; peaks at 1285 cm^−1^, 1207 cm^−1^, 1115 cm^−1^, and 1075 cm^−1^ were attributed to C-N and C-O-C stretching vibrations, C-O stretching vibration within the tertiary, and secondary and primary alcohols; peaks at 999 cm^−1^ and 865 cm^−1^ were assigned to the skeletal ring (Figure 1). The sericin spectrum revealed a specific protein spectrum with the following characteristic peaks: peak at 3291 cm^−1^ was attributed to hydroxyl stretching vibration; peak at 3070 cm^−1^ was attributed to N-H stretching vibration; peak at 2972 cm^−1^ was assigned to C-H stretching vibration; peak at 1649 cm^−1^ was assigned to C=O stretching in carbonyl group within amidic backbone (amide I); peak at 1529 cm^−1^ was attributed to N-H bending vibration and C-N stretching vibration (amide II); peak at 1395 cm^−1^ was attributed to methyl, methylene, and methine groups bending vibration; peak at 1242 cm^−1^ was attributed to C-N (amide III); peak at 1068 cm^−1^ was attributed to C-O-C and C-O stretching vibrations (Figure 1).

**Figure 2:**
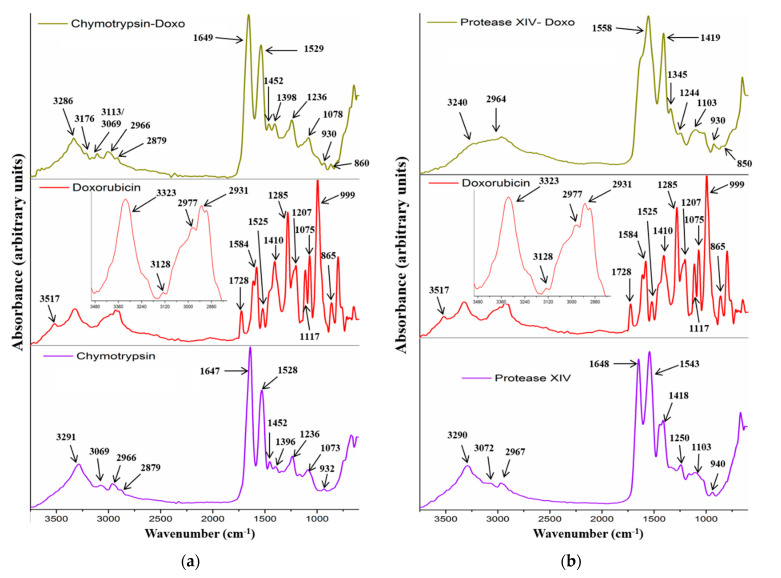
FTIR–ATR spectra of enzymes (protease IV and chymotrypsin), doxorubicin, and association of chymotrypsin/doxorubicin (**a**) and protease IV/doxorubicin (**b**).

Both enzymes (protease IV and chymotrypsin) showed a typical protein-specific spectrum with amide I, amide II, and amide III. The main peaks are shown in Figure 2a,b. Sericin–doxorubicin interaction revealed a drug-specific peak at 3510 cm^−1^, assigned to the water molecules bonded within the drug molecules. Another important peak appeared at 3070 cm^−1^, attributed to unsaturated =C-H stretching vibration characteristic for the aromatic structures similar to doxorubicin structure. Therefore, FTIR analysis showed the presence of doxorubicin within the sericin structure. Furthermore, the analysis revealed no chemical bonds between the sericin and drug, suggesting that the association was induced only by physical interactions (Figure 1).

Protease IV and chymotrypsin revealed an association with doxorubicin even to a greater extent than sericin. Thus, chymotrypsin–drug association showed two new peaks at 3176 cm^−1^ and 3113 cm^−1^, attributed to N-H stretching vibration. This fact may suggest that the amidic groups are more visible, as compared to the singular drug or enzyme. A higher contribution to the spectrum can be explained by the fact that these groups manifested supplementary physical interactions. Another important peak at 860 cm^−1^, attributed to the drug skeletal ring, appeared into the association spectrum (Figure 2a). This approach revealed that the presence of the drug molecules within the chymotrypsin structure was also governed by physical interactions. Protease IV had also some association with doxorubicin. The spectrum (Figure 2b) showed a different overview regarding drug association, as compared to the chymotrypsin approach. Therefore, the spectrum showed the absence of amide I and the presence of a broad peak centered at 1558 cm^−1^ with a significant visible shoulder around 1600 cm^−1^. This peak probably represents the contributions of amide I and amide II from the protease and peak contribution assigned to hydroxyl group bending vibration (1584 cm^−1^). The peak at 1419 cm^−1^ increased its intensity, and a new peak appeared at 1345 cm^−1^. This fact can be attributed to the contribution of the bending vibration for methyl, methylene, and methine groups. Furthermore, the presence of the peak at 850 cm^−1^, attributed to the skeletal drug structure, confirmed the presence of doxorubicin within the protease XIV structure (physical interactions). One last aspect that can be concluded from protease investigation with respect to sericin highlights the apparent stronger association interactions for the enzymes. This fact can be explained in the case of enzymes by the amide, hydroxyl, and skeletal contributions.

### 3.2. Drug Release Behavior

#### 3.2.1. Neutral Medium

**Figure 3:**
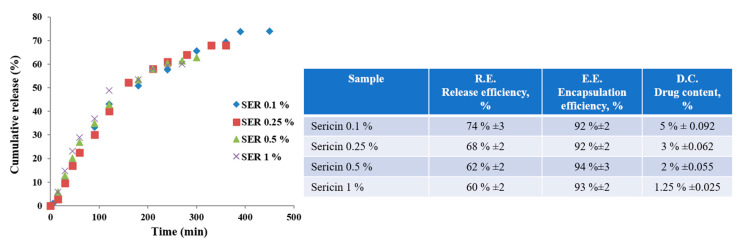
Doxorubicin release profile from sericin nanoparticles in neutral medium.

Doxorubicin release behaviors from sericin nanoparticle formulations were investigated by in vitro tests (PBS: pH 7.45, 37 °C). All nanoparticle formulations had relatively similar behavior with significant differences in release efficiency and time. The results are shown in Figure 3. Both the efficiency and the release time increased for sericin nanoparticles prepared from lower concentrations. The release profile showed a specific behavior with a faster release for the first 100 min with an efficiency of 35%. This behavior cannot be associated with a specific burst release. The release behavior followed a slower release for the next 180 min for SER 1% and for the next 360 min for SER 0.1%. Formulations with SER 0.25% and SER 0.5% followed a slower release time placed within this interval. All nanoparticles SER 0.1%–SER 1% revealed a high entrapment efficiency of 90–95%. Morphological and dimensional analyses showed that the size of the nanoparticle aggregates decreased for those obtained from lower sericin concentrations. These results may suggest that small-size aggregates reached higher efficiency but for a longer release time. The maximum cumulative release efficiency was 74%. This means there was still some entrapped doxorubicin within the mass of sericin nanoparticles. The obtained results in terms of release and encapsulation efficiency can be correlated with other sericin formulations containing low soluble agents [46,47]. Most probably, this behavior was influenced by the fact that the nanoparticles were self-assembled in the presence of doxorubicin. This approach facilitated the formation of sericin–doxorubicin conjugates which are driven by multiple physical bonding [47]. In this situation, the doxorubicin remained entrapped, and only specific environments could act as release stimulus. Therefore, specific media such as pH decrease or enzymatic activity may facilitate further release.

#### 3.2.2. Acidic Medium

**Figure 4:**
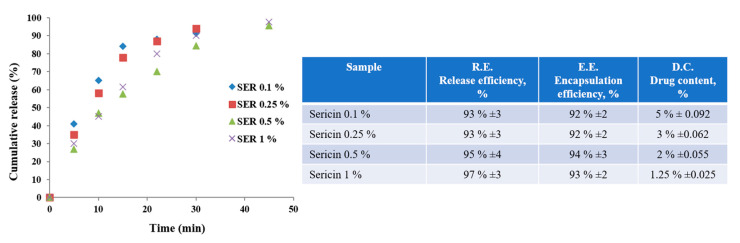
Doxorubicin release profile from sericin nanoparticles in acidic medium (pH 3).

FTIR was performed to investigate the drug–protein interaction. As previously mentioned, there are significant physical interactions that keep the drug inside the nanoparticles. Considering that the main interactions appeared between carboxyl and amino groups, a new in vitro test was performed in an acidic medium. The idea was to protonate the carboxyl negative conjugate form of COO^−^ within sericin structure to disrupt the interaction with amino positive conjugate form NH^3+^ from doxorubicin. Thus, the entrapped drug could be released. This approach was based on the sericin zeta potential results, which had a considerable negative charge into a neutral medium (pH 7.45). The analysis showed a higher release efficiency within a shorter time period, as compared to the neutral medium. Figure 4 revealed a release efficiency of over 90% for all formulations SER 0.1%–SER 1% in a release time of 30–50 min. The release profile showed different behavior with a faster release for SER 0.1% and SER 0.25% for the first 15 min, followed by a slower release for the next 15 min. SER 0.5% and SER 1% revealed a relatively constant release behavior for longer release time, as compared to SER 0.1% and SER 0.25%. This fact allowed them to reach a higher release.

#### 3.2.3. Enzymatic Media

**Figure 5:**
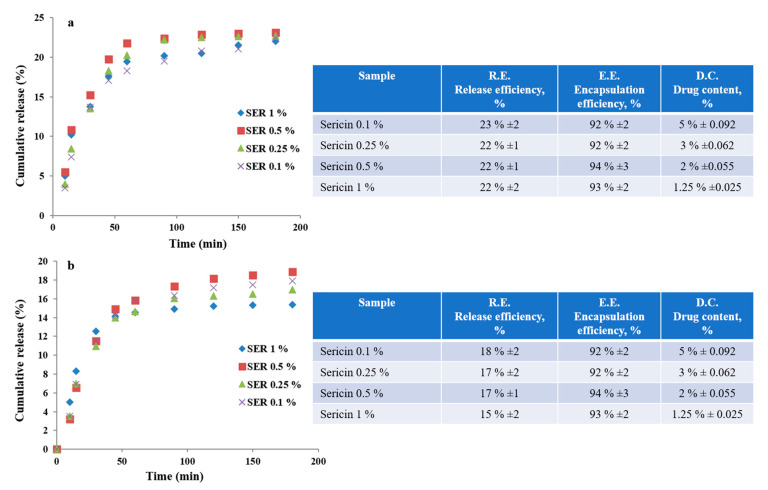
Doxorubicin release profiles from sericin nanoparticles in enzymatic media: protease XIV (**a**); chymotrypsin (**b**).

The drug–protein interaction was also investigated by enzymatic activity. Two specific enzymes, namely, protease type XIV from *Streptomyces griseus* and protease α-chymotrypsin from bovine pancreas were used. The enzymatic activity involved an enzymatic degradation of sericin protein with the easier release of entrapped drug molecules due to the disruption of physical interactions in drug–protein structure. The results showed an unexpected behavior considering the lower release with respect to the neutral medium. In the case of protease type XIV from *Streptomyces griseus*, the release efficiency was slightly over 20% (Figure 5a), while in the case of protease α-chymotrypsin from bovine pancreas, the release efficiency was slightly under 20% (Figure 5b). Both situations followed a similar profile release and a release time of 180 min. The profile showed a faster release within the first 50 min, followed by a slower release. The poor release efficiency can be explained by the strong physical interactions between the doxorubicin molecule and enzymes’ chemical structure. In this case, the drug molecules released from the nanoparticles’ mass were further embedded in the structure of the enzymes. These results are in good agreement with the FTIR analysis that showed stronger interactions within drug–enzyme association as compared to sericin nanoparticles.

### 3.3. Morphological Characterization

#### 3.3.1. SEM Analysis

**Figure 6:**
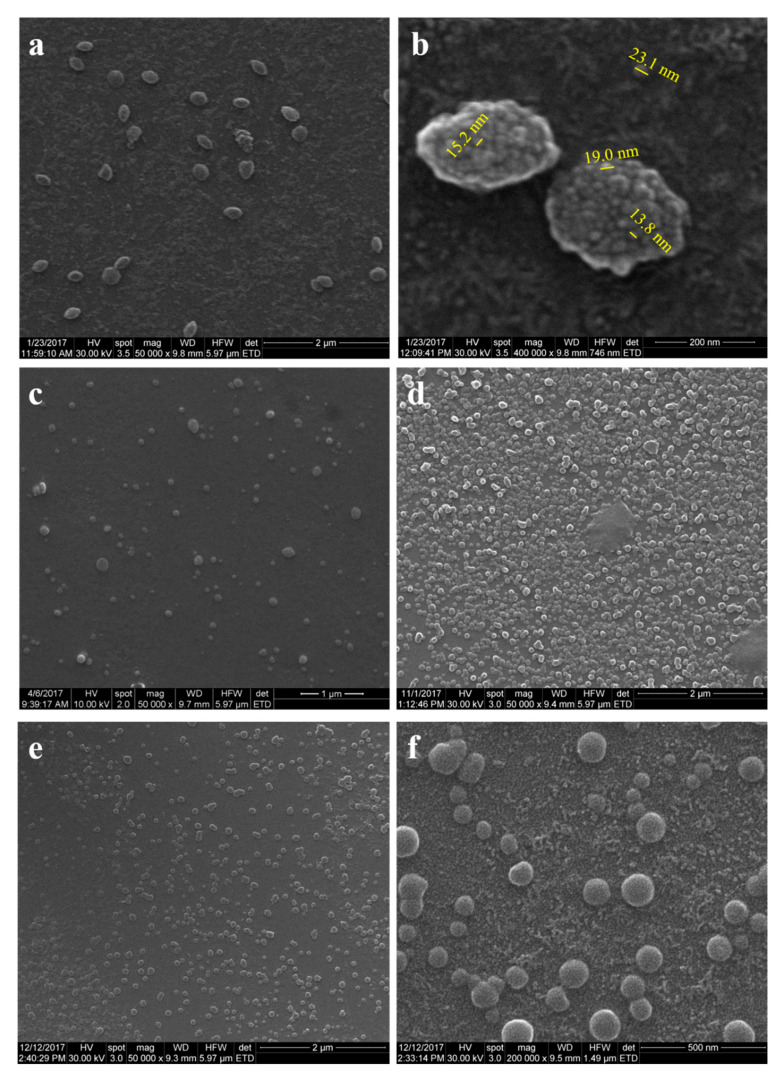
SEM microphotographs for sericin nanoparticles from various sericin concentrations: (**a**,**b**) SER 1%; (**c**,**d**) SER 0.5% and SER 0.25%; (**e**,**f**) SER 0.1%.

SER 1% nanoparticles were obtained with a size distribution of 200–300 nanometers with round and specific fusiform shapes (Figure 6a). Higher magnification images revealed closer insights into nanoparticles’ morphology with a bunch nanostructure and nanowaved surface (Figure 6b). The nanowaved morphology showed lower sizes of 15–20 nm winding the surface. Individual nanoparticles of 23 nm could be detected, suggesting that the bunch nanostructuring is formed of aggregates of smaller nanoparticles. Morphological characterization continued with formulations SER 0.5% and SER 0.25%. The results for SER 0.5% and SER 0.25% formulations revealed nanoparticle aggregates with a size range size of 100–200 nm (SER 0.5%) and 100–150 nm (SER 0.25%), as shown in Figure 6c,d. Aggregates for SER 0.5% had round and fusiform shapes (Figure 6c), while those for SER 0.25% exhibited only round shapes (Figure 6d). This could be explained by the size range differences since the size increase led to the deviation from the usual round shape. Formulation SER 0.1% was investigated in order to reveal a specific trend of sericin formulations. The lower the sericin concentration was, the smaller the nanoparticle aggregates were obtained. Figure 6e showed aggregates with a size range of 80–130 nm. The aggregates had a round shape like SER 0.25%. The higher magnification image revealed the same bunch nanostructuring with a nanowaved surface (Figure 6f).

#### 3.3.2. TEM Analysis

**Figure 7:**
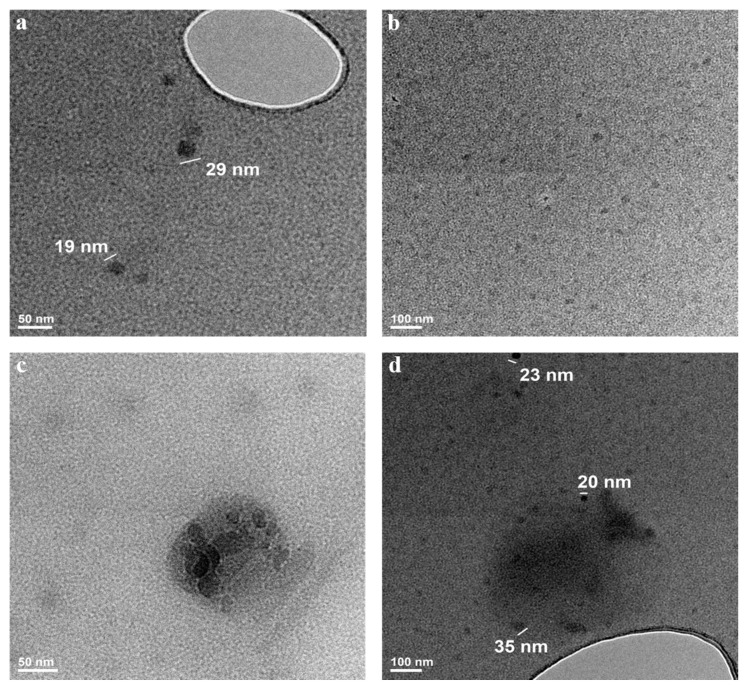

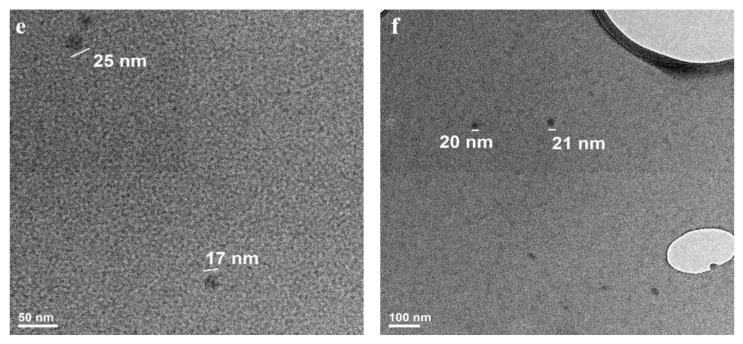
TEM images for individualized nanoparticles obtained from 1% sericin concentration (**a**,**b**); TEM images for nanoparticles aggregates obtained from 1% sericin concentration (SER 1%) (**c**,**d**); TEM images for individualized nanoparticles obtained from 0.1% sericin concentration (SER 0.1%) (**e**,**f**).

TEM analysis was performed to confirm the SEM results regarding aggregates size, morphology, and nanostructuring. Formulation SER 1% exhibited individualized nanoparticles with a range size between 20 and 35 nm (Figure 7a, higher magnification). The overview image showed also individualized nanoparticles in a significant number (Figure 7b). The nanoparticle aggregates revealed the internal nanostructuring. The aggregates were composed of smaller nanoparticles of 20–35 nm (Figure 7c,d). Besides aggregates, one may notice individualized nanoparticles, suggesting that only a part of them associate with such structures. Formulation SER 0.1% had even smaller individualized nanoparticles with respect to other formulations (15–25 nm, Figure 7e,f). This result confirmed the formulation trend with smaller nanoparticles for lower sericin concentration. This fact can explain the size differences between the aggregates or their shape. The nanoprecipitation appeared as a suitable self-assembling method able to optimize the nanoparticles’ features among other nanoformulation methods [30,48,49]. The nanoparticle low average dimension was directly related to some important sericin properties. The mechanism followed a nucleation step, which is typical for the nanoprecipitation method [37,50,51,52]. This mechanism assured the preparation of a high number of small nanoparticles.

### 3.4. Dynamic Light Scattering, Zeta Potential, and Isoelectric Point

**Figure 8:**
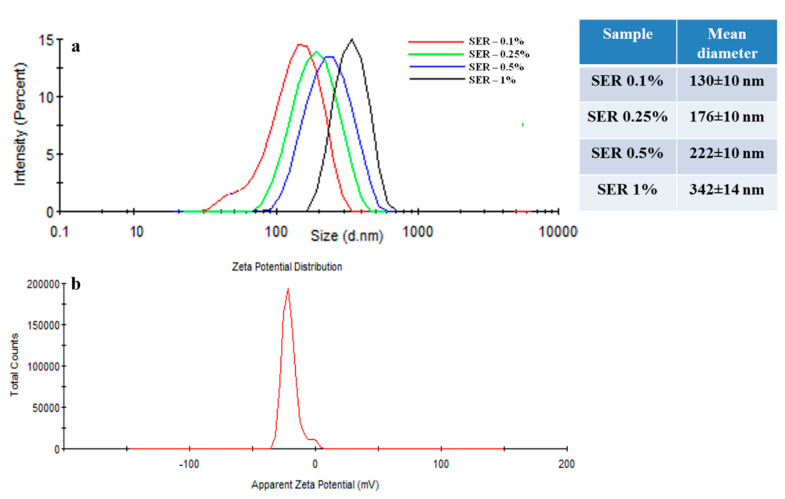
DLS dimensional distribution of sericin nanoparticles with various concentrations (**a**); DLS zeta potential of sericin nanoparticles—SER 0.1% formulation (**b**).

DLS analysis showed more specifically nanoparticles aggregates size. The sericin nanoparticle formulations SER 0.1%–SER 1% revealed an increase of the mean diameter with the increase of the concentration of sericin solutions. All formulations showed a close size distribution profile and close mean size diameter. The size distribution of sericin nanoparticle aggregates and mean diameter are shown in Figure 8a. The results confirmed the morphological investigation by SEM and TEM on aggregates size increasing profile but with a higher size distribution due to the swelling effect.

The nanoparticles’ surface zeta potential and isoelectric point were used to evaluate the protein surface charging at various pH values. The results revealed a negative surface charging with a zeta potential of −20.2 mV with a standard deviation ±0.9 mV (Figure 8b.) This value represents the zeta potential for neutral pH (7.45). The isoelectric point was established in the pH range 2–2.5. This value of zeta potential can influence the tumor cell line interaction or the mechanism of nanoparticle synthesis.

#### Molecular Weight Evaluation by DLS

The average molecular weight of silk sericin was evaluated by the detection of light scattering based on the interactions of protein molecule–light. Therefore, the sericin solutions were exposed to a monochromatic wave of light and using multiple detectors. The analysis supposed the investigation of four diluted solutions with various concentrations between 0.2 and 2% (*w*/*v*) of silk sericin. An average molecular weight of about 11,700 ± 100 g/mole was determined.

### 3.5. Conformational Analysis by Circular Dichroism (CD)

**Figure 9:**
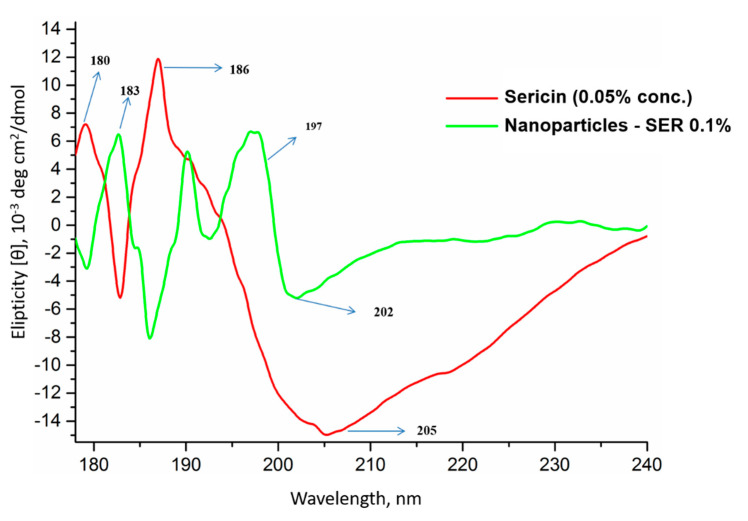
CD curves of native sericin (0.05 wt.% concentration) and sericin nanoparticles (SER-0.1%).

CD analysis for sericin showed two positive peaks and one sharp negative peak. The positive peaks at 180 and 186 nm, together with the negative peak at 205 nm, suggest a secondary conformational arrangement dominated by β-sheet and random coil. The sericin nanoparticles had a shifting of the negative peak to lower values and shifting of the positive peaks to higher values. The positive peak was also split into two peaks (Figure 9). This shows some conformational changes of random coil toward the β-sheet structure.

The sericin formulations led to the obtaining of sericin nanoparticles ranging between 15 and 40 nm depending on the solution concentration. These size values are below the usual sizes of polymeric nanoparticles. This fact can be attributed to several important factors including sericin chemistry, molecular weight, concentration, or preparation method. In the case of molecular weight (MW), there are various studies in the literature showing the influence or not of the MW on the size of the nanoparticles for different polymeric systems. Most probably, the molecular weight’s influence on nanoparticles size is directly correlated to every studied system. In our study, the low molecular weight fitted the sericin in the oligomeric range (polypeptides), and it could induce such small nanoparticles. The sericin chemistry clearly influenced the hydrophilicity, water solubility, or behavior within the organic phase (acetone). The high water solubility could be also influenced by the low molecular weight. The high water solubility, together with the low molecular weight and chemistry, positively influenced the sericin behavior in acetone dispersion. In contact with acetone, the sericin molecules gather and induce a nucleation process. This is a more controlled process mechanism than instant precipitation. This approach is directly correlated to the preparation method. Nanoprecipitation follows a three-stage process: nucleation, growth, and aggregation [52,53,54]. Therefore, this approach allowed a supersaturation of sericin molecules per volume with a nucleating process, followed by a growth step. A high sericin concentration led to the generation of nanoparticles with a larger size distribution reaching 35–40 nm, while a low concentration led to the generation of nanoparticles with a narrow distribution. Both high (1% *w*/*v*) and low (0.1 *w*/*v*) concentrations led to the generation of a relatively high number of nanoparticles, suggesting no influence on the number of nanoparticles. Thus, in the initial stage, a high number of nanoparticles were formed, while the size differences appeared in the growth step due to the addition of new molecules on the nuclei surface. Another important issue to be addressed is the aggregation process. The dimensional and morphological investigation showed larger nanoparticle aggregates with a range size between 100 and 300 nm. These nanoparticle aggregates were formed along with individual nanoparticles. Therefore, this process probably appears only in the case of supersaturation for nanoparticles’ concentration per volume of dispersion media.

### 3.6. In Vitro Antitumor Activity Evaluation of DOX-Loaded Sericin Nanocarriers

**Figure 10:**
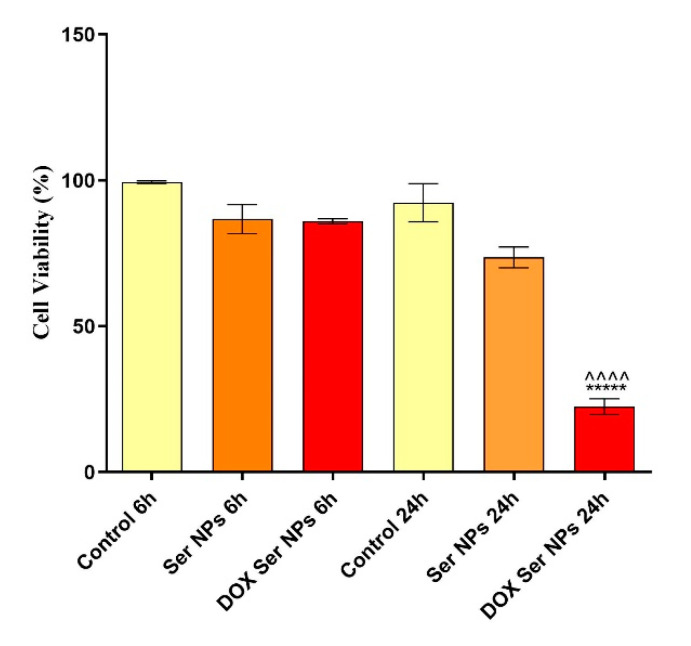
Graphical representation of MCF-7 breast cancer cells viability after 6 h and 24 h of treatment with free and DOX-loaded Ser NPs (Dox Ser NPs vs. untreated control **** *p* ≤ 0.0001; Dox Ser NPs vs. Ser NPs ^^^^ *p* ≤ 0.0001).

As described above, based on the nanoparticles’ size and doxorubicin release profile, the 0.1% sericin formulation was employed in the in vitro biological investigations. To evaluate the viability of MCF–7 breast cancer cells after 6 h and 24 h of exposure to unloaded Ser NPs and DOX-loaded Ser NPs, the quantitative MTT assay was performed. Data were statistically analyzed and graphically represented in Figure 10 using GraphPad Prism 6 software.

**Figure 11:**
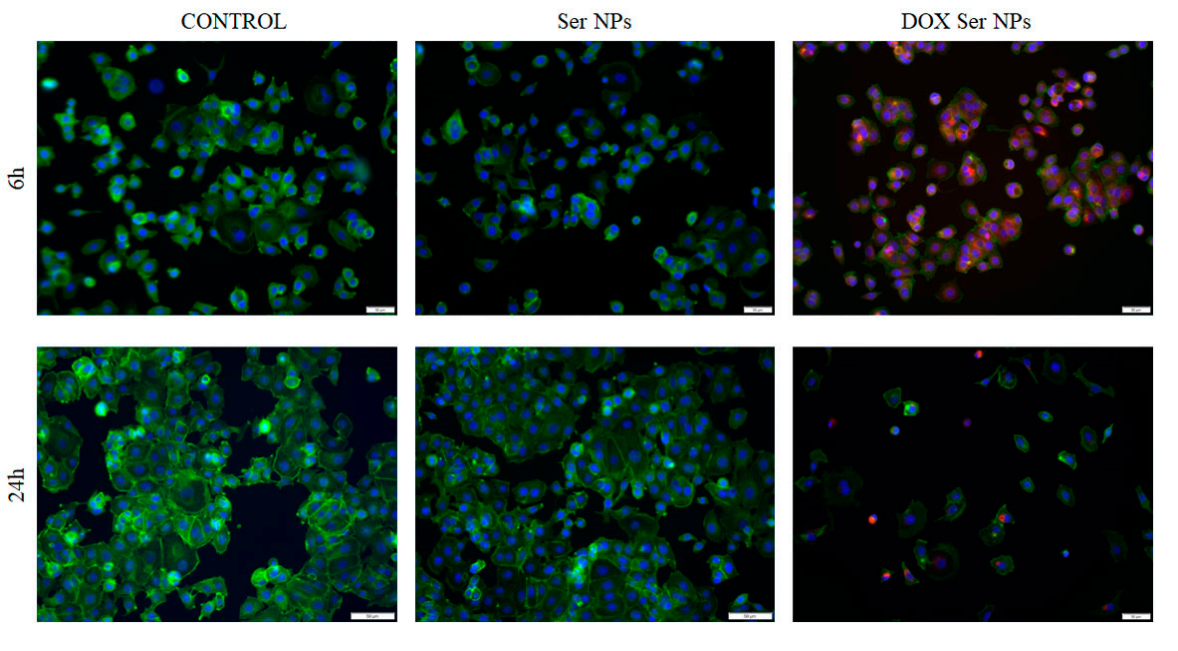
Fluorescence microscopy images of MCF-7 cells treated for 6 h and 24 h with free Ser NPs and DOX-loaded Ser NPs, as compared with an untreated MCF-7 monolayer: green fluorescence–phalloidin–FITC: actin filaments; blue fluorescence–DAPI: cell nuclei; red fluorescence–DOX). Scale bar: 50 μm.

Our data showed that after 6 h of treatment, none of the treatments induced cell viability alterations. Moreover, after 24 h of exposure to free Ser NPs, the viability of the MCF-7 cells remained similar to the control, demonstrating good biocompatibility of the pristine sericin nanocarriers. In contrast, after 24 h of treatment, the DOX-loaded Ser NPs significantly decreased the viability of the MCF-7 cells (**** *p* ≤ 0.0001). Moreover, MCF-7 breast cancer cell morphology was investigated by fluorescence microscopy after staining the cytoskeleton fibers with phalloidin–FITC and the cell nuclei with DAPI. The images captured are presented in Figure 11. No alterations were produced by the treatment with unloaded Ser NPs during 24 h, as compared with the untreated cells. In contrast, the treatment with DOX-loaded Ser NPs induced modifications in terms of actin filaments’ organization and distribution in the cellular cytoplasm. Additionally, the fluorescence microscopy images captured in the samples treated with DOX-loaded Ser NPs revealed red fluorescence inside the MCF-7 cells. Considering that doxorubicin is well known as a red fluorescent chemical compound, this valuable observation indicates/proves that the DOX-loaded Ser NPs successfully enter the cells.

**Figure 12:**
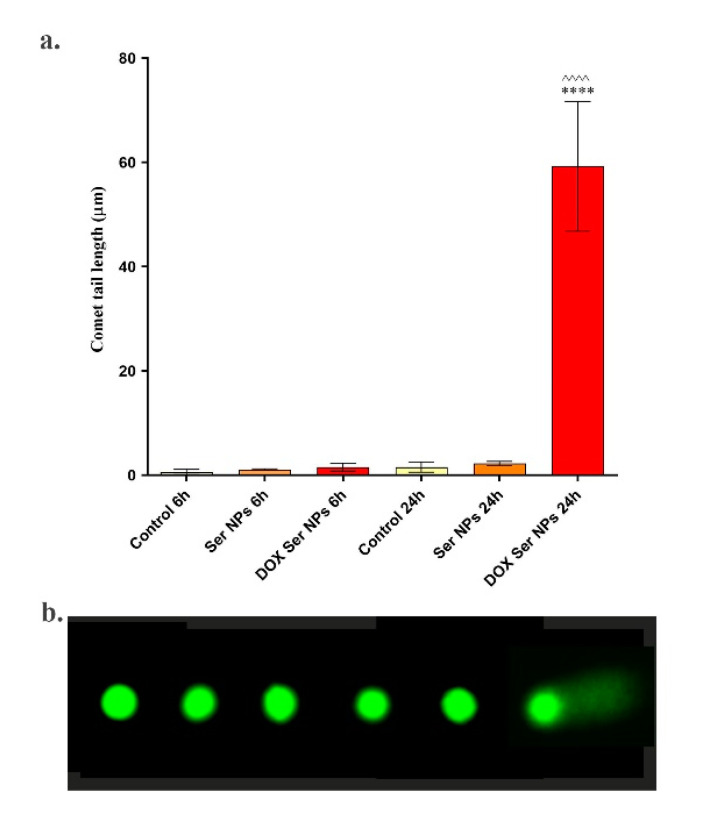
(**a**) Graphical representation showing the comet tail length as an indicator of DNA damage in MCF–7 cell cultures treated for 6 h and 24 h with simple and DOX-loaded Ser NPs (Dox Ser NPs vs. untreated control **** *p* ≤ 0.0001; Dox Ser NPs vs. Ser NPs ^^^^ *p* ≤ 0.0001); (**b**) representative fluorescence micrographs of the comet-like structures in MCF–7 cell cultures treated for 6 h and 24 h with simple and DOX-loaded Ser NPs.

Finally, to assess the genotoxic potential of the DOX-loaded Ser NPs treatment, the comet assay was performed. After fluorescence image processing and data analysis, the DNA damage profile in MCF-7 cancer cells after Ser NPs + DOX treatment was established based on the average length of the comet tails. As presented in Figure 12, the comet-like structures correlated with enhanced DNA migration were identified only in MCF-7 cells exposed for 24 h to the treatment with DOX-loaded Ser NPs. As DNA damage is a hallmark of apoptosis, our data suggest that DOX encapsulation in Ser NPs triggers apoptosis of MCF–7 breast cancer cells.

Sericin nanocarriers have previously been used for breast cancer management [28]. Mandal and Kundu showed that paclitaxel-loaded sericin nanocarriers induced apoptosis in MCF-7 breast cancer cells. Similarly, we demonstrated that DOX-loaded Ser NPs significantly decreased MCF-7 cells viability after 24 h of treatment and altered the morphology of the cells, as revealed by the fluorescent labeling of the cell’s cytoskeleton.

Regarding DOX, the literature reports two potential mechanisms of action in the cancer cell: (i) the intercalation into DNA and disruption of topoisomerase-II-mediated DNA repair and (ii) the generation of free radicals producing damages to cellular membranes, DNA, and proteins [55]. Our data showed that the DOX-loaded Ser NPs induced DNA damage in MCF-7 cells, as compared with the pristine Ser NPs, probably due to the toxic effect of the delivered DOX.

## 4. Conclusions

In conclusion, we obtained sericin nanoparticles with a size range between 15 and 40 nm. This dimensional range is below the usual range of the polymeric nanoparticles. The nanoprecipitation proved to be a suitable method for sericin nanoparticles’ preparation, loading, and release. The advanced morphological investigation showed the size and size distribution of the nanocarriers with a direct positive influence on the biological investigation. Moreover, we also showed that the DOX-loaded Ser NPs significantly decreased MCF-7 cells viability, altered their morphology, and induced DNA damage, as compared with the unloaded Ser NPs.

## Data Availability

The data presented in this study are available on request from the corresponding author.
